# Simultaneously Screening for Liver Steatosis and Fibrosis in Romanian Type 2 Diabetes Mellitus Patients Using Vibration-Controlled Transient Elastography with Controlled Attenuation Parameter

**DOI:** 10.3390/diagnostics12071753

**Published:** 2022-07-20

**Authors:** Anca Trifan, Ermina Stratina, Robert Nastasa, Adrian Rotaru, Remus Stafie, Sebastian Zenovia, Laura Huiban, Catalin Sfarti, Camelia Cojocariu, Tudor Cuciureanu, Cristina Muzica, Stefan Chiriac, Irina Girleanu, Ana-Maria Singeap, Carol Stanciu

**Affiliations:** 1 Department of Gastroenterology, Grigore T. Popa University of Medicine and Pharmacy, 700115 Iasi, Romania; ancatrifan@yahoo.com (A.T.); adrianrotaru94@yahoo.com (A.R.); stafieremus@gmail.com (R.S.); sebastianzenovia20@gmail.com (S.Z.); huiban.laura@yahoo.com (L.H.); cvsfarti@gmail.com (C.S.); cameliacojocariu@yahoo.com (C.C.); drcuciureanutudor@gmail.com (T.C.); lungu.christina@yahoo.com (C.M.); stefannchiriac@yahoo.com (S.C.); gilda_iri25@yahoo.com (I.G.); anamaria.singeap@yahoo.com (A.-M.S.); stanciucarol@yahoo.com (C.S.); 2 Institute of Gastroenterology and Hepatology, “St. Spiridon” Emergency Hospital, 700111 Iasi, Romania

**Keywords:** type 2 diabetes mellitus, non-alcoholic fatty liver, vibration-controlled transient elastography

## Abstract

Non-alcoholic fatty liver disease (NAFLD) is a common finding among patients with type 2 diabetes mellitus (T2DM). Between NAFLD and T2DM exist a bidirectional relationship. Patients with T2DM are at high risk for NAFLD, and evidence suggests that T2DM is linked to progressive NAFLD and poor liver outcomes. NAFLD promotes the development of T2DM and leads to a substantial increase in the risk of T2DM complications. This study aimed to assess the prevalence of liver steatosis and fibrosis in patients with T2DM from north-eastern Romania by using Vibration-Controlled Transient Elastography (VCTE) with Controlled Attenuation Parameter (CAP), which is a non-invasive method and can assess simultaneously liver steatosis and fibrosis. In total, 424 consecutive patients with T2DM were enrolled and evaluated using VCTE with CAP from January 2020 to January 2022. Clinical and laboratory data were recorded in all patients. For the CAP score, we used the following cut-offs: mild steatosis (S1)—274 dB/m, moderate steatosis (S2)—290 dB/m, and severe steatosis (S3)—302 dB/m. For liver fibrosis, to differentiate between fibrosis stages, the cut-off values were F ≥ 8.2 kPa for significant fibrosis (F2), F ≥ 9.7 kPa for advanced fibrosis (F3), and F ≥ 13.6 kPa for cirrhosis (F4). In total, 380 diabetic patients (72.6%) had liver steatosis (51.3% females, the mean age of 55.22 ± 10.88 years, mean body mass index (BMI) 29.12 ± 5.64 kg/m^2^). Among them, 26 (8.4%) patients had moderate liver steatosis (S2) and 242 (78.5%) patients had severe hepatic steatosis (S3). According to VCTE measurements, 176 (57.14%) patients had liver fibrosis, 36 (11.7%) of them had advanced fibrosis (F3), and 42 (13.6%) diabetic patients had cirrhosis (F4). Univariate analyses showed that severe steatosis was significantly associated with ferritin (β = 0.223, *p* = 0.022), total cholesterol (β = 0.159, *p* = 0.031), and HDL-cholesterol (β = −0.120, *p* = 0.006). In multivariate analyses, BMI (β = 0.349, *p* < 0.001), fasting plasma glucose (β = 0.211, *p* = 0.006), and triglycerides (β = 0.132, *p* = 0.044) were predictors of S3. Patients with T2DM have a high prevalence of severe steatosis and advanced fibrosis which can lead to the development and progression of complications with high morbidity and mortality rates. Hence, it is necessary to implement screening strategies to prevent advanced liver disease in patients with T2DM.

## 1. Introduction

Type 2 diabetes mellitus (T2DM) had an increasing prevalence in the past decades in parallel with the prevalence of obesity and metabolic syndrome (MetS) [1]. Non-alcoholic fatty liver disease (NAFLD) and T2DM have a bidirectional relationship; NAFLD leads to insulin resistance (IR) and T2DM through different mechanisms, but T2DM also induces the progression of liver disease as an independent risk factor. NAFLD is a common finding among patients with T2DM and encompasses a spectrum of pathological conditions, which range from simple steatosis (NAFL) to non-alcoholic steatohepatitis (NASH), and cirrhosis and hepatocellular carcinoma [2,3]. NAFLD is considered the hepatic component of MetS and its characteristics are similar to other metabolic disorders such as obesity and T2DM. Individuals with T2DM have a prevalence of NAFLD at approximately 75%, which is more than the prevalence in non-diabetic patients. Moreover, the patients with NAFLD have an increased risk of developing T2DM at least twice as high as those without NAFLD, and patients with severe steatosis had a greater risk of developing T2DM. The risk of all-cause mortality is increased by 2.2—a fold risk in patients with NAFLD and T2DM [4].

NAFLD and T2DM have common physiopathological pathways represented by hyperinsulinemia, systemic inflammation, IR, oxidative stress, and excessive production of free fatty acids (FFA) [5]. IR is the principal link between NAFLD and T2DM. IR is defined as a suboptimal activity of insulin in different tissues [6]. IR leads to decreased transport of FFA to the liver, decreased B-oxidation of FFA, increased de novo lipogenesis, and increased triglyceride synthesis [7]. Consequently, the beta cells of the pancreas will be secreting more insulin. In patients with T2DM insulin, clearance is also suppressed. All these mechanisms lead to hyperinsulinemia, which causes hepatocellular ballooning and lobular inflammation with the progression of NAFLD to NASH in T2DM patients [8].

Liver damage was underestimated among diabetic patients, but in the last decades, many studies underlined the severity of liver disease in these patients [9]. T2DM, obesity, and other components of MetS can lead to the deposit of lipid droplets in hepatocytes, promoting NAFLD. If the inflammation occurs, NAFLD progress to non-alcoholic steatohepatitis (NASH) with progressive fibrosis [10]. In studies from Turkey and France, the prevalence of advanced fibrosis was more important in patients with T2DM than in those without T2DM [11,12].

Regarding diagnostics modalities of NAFLD among patients with T2DM, several studies used liver biopsy (LB) as a reference standard, principally for the diagnosis of NASH [13]. LB represents an invasive method, with many outcomes, such as sampling errors, intra- and inter-observer variability, limited accessibility, and difficult patient acceptance [14,15]. Abdominal ultrasonography (US) is a first-line non-invasive imaging test for assessing liver steatosis detection. It has several disadvantages, such as low sensitivity, and can detect moderate and severe steatosis [16].

Vibration-controlled transient elastography (VCTE) (FibroScan^®^, EchoSens, Paris, France) consists of measuring the velocity of a 2.5 to 3.5 MHz shear wave passing through the liver and generating the stiffness score measured in kPa. It has a sensitivity of 100%, specificity of 73.9%, and accuracy of 86.4% in identifying fibrosis in NAFLD [17,18,19]. Additionally, it is a fast method, simple to be performed, repeated, and reproduced, being widely used in clinical practices [20]. The addition of controlled attenuation parameter (CAP), used for the assessment and quantification of liver infiltration with lipids, makes FibroScan a valuable system for hepatic assessment in patients at risk for developing NASH [21,22]. From two decades ago when the medical applicability of the shear elasticity probe was introduced, based on the reflection mode for evaluation of the soft tissues (breast, thyroid, prostate, or liver), the pathological state of them was correlated with changes in stiffness [23,24]. This explains the great interest that persisted over the years in the development of multiple elastography techniques, which have become a state of the art in stadialization of liver fibrosis as steatosis.

Herein, we aimed to evaluate the prevalence of liver steatosis and fibrosis among patients with T2DM using the VCTE and CAP score. Another aim of our study consists of identifying the risk factors associated with hepatic steatosis and fibrosis in individuals with T2DM.

## 2. Materials and Methods

### 2.1. Patients

In total, 424 patients diagnosed with T2DM agreed to be evaluated by VCTE measurements. The subjects were referred to our clinic by general practitioners and colleagues in other specialties, were prospectively enrolled from January 2020 to January 2022 at the Gastroenterology and Hepatology Institute, in Iasi, Romania. Exclusion criteria were patients with other chronic liver diseases such as hepatitis B/C, hemochromatosis, Wilson’s disease, alcohol consumption more than 20 g/day in women and >30 g/day in men, use of steatogenic medication, pregnancy, malignancy, heart failure, end-stages renal diseases, the elevation of aspartate liver enzymes more than five times the upper limit of normal (ULN) values, and unreliable VCTE and CAP measurements.

The study was performed following the principles of the Declaration of Helsinki and was approved by the Ethics Committee of our Institute. All participants signed informed written consent forms.

### 2.2. Vibration-Controlled Transient Elastography (VCTE) and Controlled Attenuation Parameter (CAP) Measurements

The patients included in our study were examined for liver fibrosis and steatosis using the FibroScan^®^ 502 touch model (Echosens, Paris, France) equipped with the M-(standard) or XL-(obese) probe by a single operator. After at minimum four hours of fasting, patients were examined in the supine position with the right arm in maximum abduction, leading to a better intercostal window to the right lobe liver scanning. Firstly, the examination was performed using the M-probe with a transducer frequency of 3.5 MHz. The XL-probe (2.5 MHz) was used at indication of machine if the distance between the skin-to-liver capsule was higher than 25 mm. Reliable measurement was considered if 10 acquisitions were made with an interquartile interval not higher than 30%. CAP is a quantitative method and is measured in decibel-milliwatts (dB/m). Regarding the cut-offs for CAP values, they were as follows: mild steatosis (S1)—274 dB/m, moderate steatosis (S2)—290 dB/m, and severe steatosis (S3)—302 dB/m. Regarding the cut-offs for LSM, we used the following values: F2 ≥ 8.2 kPa, F3 ≥ 9.7 kPa, and F4 ≥ 13.6 kPa [25].

### 2.3. Clinical and Laboratory Assessment

Patients had a complete clinical examination with laboratory tests and underwent VCTE examination on the same day. Demographic and clinical data were collected including sex, age, daily alcohol intake, tobacco use, BMI, waist circumference, type of antidiabetic treatments, and systolic and diastolic blood pressure. The following laboratory data were collected: hemoglobin, international normalized ratio (INR), fibrinogen, alanine aminotransferase (ALT), aspartate aminotransferase (AST), gamma-glutamyl transpeptidase (GGT), alkaline phosphatase (ALP), bilirubin, albumin, total proteins, urea, serum creatinine, total cholesterol, triglycerides, low-density lipoprotein (LDL-c), high-density lipoproteins (HDL-c), and fasting blood glucose. BMI was calculated using the formula weight in kilograms divided by square of height in meters. Overweight and obese were defined as having BMI between 25 and 30 kg/m^2^ and BMI ≥ 30 kg/m^2^, respectively [26].

### 2.4. Statistical Analysis

All statistical tests were performed using IBM SPSS, Version 22.0 (IBM SPSS Inc, Chicago, IL, USA). Qualitative variables are expressed as numbers (percentage). Baseline characteristics and clinical variables were expressed as mean ± standard deviation if normally distributed. If not normally distributed, they were expressed as median (25th and 75th percentiles). Distribution analysis was performed using the Kolmogorov–Smirnov test. Parametric tests, such as t-test and ANOVA, were used for the assessment of differences between numerical variables with normal distribution. Non-parametric tests, such as Mann–Whitney or Kruskal–Wallis tests, were used for variables with non-normal distribution. Univariate linear regression was performed to assess the factors that may influence the CAP and LSM values, followed by multivariate linear regression using only the significant factors. Pearson correlation coefficient (r) was used for establishing the association between two variables. A *p*-value of < 0.05 (two-tailed) was considered statistically significant.

## 3. Results

### 3.1. Characteristics of the Study Population

**Figure 1:**
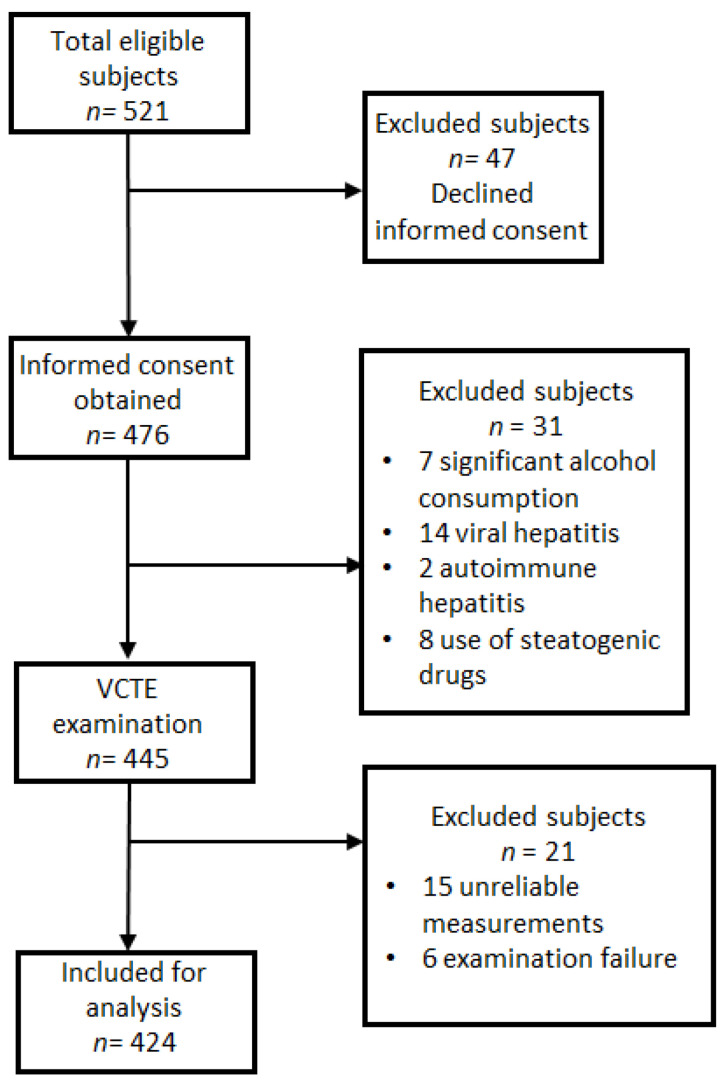
Participant flowchart. A total of 97 subjects were ruled out from the study.

Of the total of 521 eligible subjects, 476 accept to participate in the study and were screened using VCTE and CAP (Figure 1). A total of 52 patients were excluded: 14 patients for the presence of antibodies anti-HVC or the presence of AgHbs, 7 patients due to the significant alcohol consumption, 2 patients were diagnosed with autoimmune hepatitis, and 8 patients who used steatogenic drugs in the last year. Moreover, a total of 21 patients were excluded from the final analysis, because of unreliable measurements in 15 cases and examination failure in 6 cases.

In the final analysis, we included 424 patients who accomplished the admission criteria. Of these patients with a mean age of 53.67 ± 11.37 years, 331 (78.1%) of them were evaluated by the M probe, and 93 (21.9%) were evaluated by the XL probe. All baseline characteristics are presented in Table 1. Most of the participants had a BMI ≥ 25 kg/m2 (84.8%) with a higher number of female participants (51.7%). Hypertension and metabolic syndrome were present in 246 (58%) and 229 (54%) patients, respectively.

Out of 424 T2DM screened subjects, the median LSM value was 7.48 ± 5.0111 kPa, and according to VCTE measurements, 182 (43%) of them had no fibrosis-F0, 110 (25.9%) had mild fibrosis-F1, 34 (8.1%) had significant fibrosis-F2, 52 had advanced fibrosis (12.3%), and 45 (10.7%) had cirrhosis (F4). Individuals with CAP score ≥ 274 dB/m were elderly (mean age 55.22 ± 10.88 years), with a higher BMI (mean value of 29.12 ± 5.64 kg/m2), and presented liver cirrhosis at a percentage of 13.6% vs. 2.6% for patients without steatosis. Additionally, the proportion of abdominal obesity is significantly increased among patients with steatosis, of whom 71.1% had abdominal obesity. 

In the study cohort, the distribution of steatosis degree was as follows: 40 (13.1%) had S1, 26 (8.4%) S2, and 242 (78.5%) patients had S3 (Table 1). Moreover, according to VCTE measurements, 69.9% (392 patients) had no or mild fibrosis—F0 and F1, 8.1% (34) had F2, 12.3% (52) had F3, and 8.2% (43 patients) had F4, with a higher LSM value among patients with steatosis (mean LSM 8.02 ± 5.16 kPa vs. 6.32 ± 4.74 kPa, *p* < 0.001). There was no significant difference in fibrosis severity by VCTE according to steatosis degree (Table 1).

### 3.2. Characteristics of Patients According to Liver Fibrosis Stage

Out of 424 screened subjects, the median LSM value was 7.48 ± 4.11 kPa, and according to VCTE measurements, 98 (23.1%) had at least advanced fibrosis (≥F3), which was evaluated by the M probe, obtaining a percentage of 62.2%. Most of the participants with an LSM value ≥ 9.7 kPa had a BMI ≥ 25 kg/m^2^ (81.7%), with the percentage of male and female gender approximately equal (51% females). Moreover, these individuals were elderly (mean age 55.15 ± 12.24 years, *p* = 0.041) and presented severe degrees of steatosis (*p* = 0.008) in comparison with those with no-to-moderate fibrosis (≤F2), with a mean CAP score of 319.54 ± 45.47 dB/m. According to laboratory data, patients with at least advanced fibrosis (≥F3) had higher values of AST (*p* < 0.001), fasting plasma glucose (*p* = 0.024), triglycerides (*p* = 0.047), serum uric acid (*p* < 0.001), and AFP (*p* = 0.021). Additionally, individuals with LSM value ≥ 9.7 kPa had decreased values of platelet count (*p* < 0.001), albumin (*p* < 0.001), and HDL-C (*p* = 0.004) (Table 2). Although the presence of hypertension was more often found in individuals with an LSM < 9.7 kPa (60.4% vs. 50% respectively), there was no significant difference among the groups (*p* = 0.072).

### 3.3. Factors Associated with Increased LSM Values

We performed a univariate linear regression analysis to identify factors associated with increased LSM and CAP values, and only those with a significant *p*-value (*p* < 0.05) were included in the multivariate regression analysis (Table 3). In the univariate analysis, we found that age (β = 0.123, *p* = 0.004), BMI (β = 0.096, *p* < 0.001), platelets (β = −0.307, *p* < 0.001), fasting plasma glucose (β = 0.121, *p* = 0.002), triglycerides (β = 0.177, *p* = 0.001), alanine aminotransferase (ALT) (β = 0.297, *p* = 0.085), aspartate aminotransferase (AST) (β = 0.306, *p* < 0.001), and serum uric acid (β = −0.17, *p* = 0.016) were risk factors associated with LSM value in all patients. From these factors, in the multivariate analysis, only age (β = 0.157, *p* = 0.013) and BMI (β = 0.142, *p* = 0.013) were independently associated with increased LSM value (Table 3). 

### 3.4. Factors Associated with an Increased CAP Score

**Figure 2:**
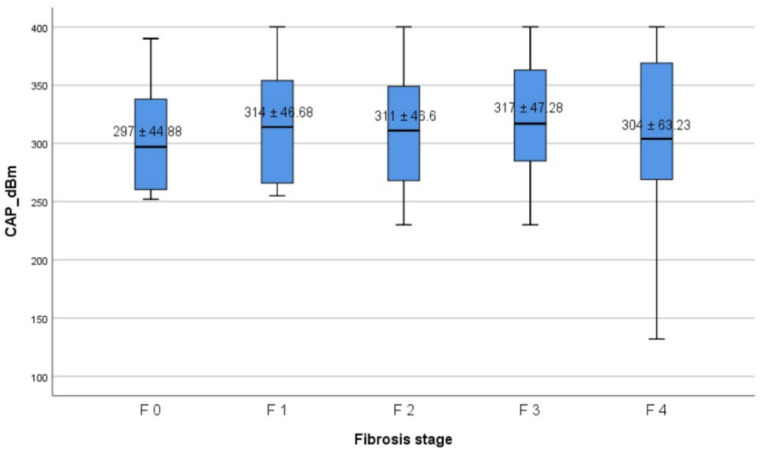
CAP values according to fibrosis stages. The bottom and the top of each box represent the 25th and 75th percentiles, while the lines through the box indicate the median.

We noticed that ferritin (β = 0.223, *p* = 0.022), total cholesterol (β = 0.159, *p* = 0.031), and HDL-cholesterol (β = −0.120, *p* = 0.006) were risk factors associated only with the CAP score in the univariate analysis, but in the multivariate analysis, none of them are associated with the CAP score. However, in the entire cohort, we found that BMI (β = 0.349, *p* < 0.001), fasting plasma glucose (β = 0.211, *p* = 0.006), and triglycerides (β = 0.132, *p* = 0.044) were independent risk factors associated with an elevated CAP score on multivariate linear regression analysis. Therefore, BMI remains the only independent risk factor that is strongly associated with both CAP and LSM values (Table 4). Furthermore, according to fibrosis grades, the median value of CAP was 297 ± 44.48 dB/m in patients without fibrosis (F0), 314 ± 46.68 dB/m in mild fibrosis (F1), 311 ± 46.6 in significant fibrosis (F2), 317 ± 47.28 dB/m in advanced fibrosis (F3), and 304 ± 63.23 dB/m in patients with cirrhosis (F4) (Figure 2). Additionally, there was a significant increasing of LSM (*p* < 0.001) and CAP values between fibrosis stages (*p* = 0.043) with decreased CAP values in cirrhotic patients (Table 5).

## 4. Discussion

NAFLD has become one of the most common chronic liver diseases worldwide and a major public health concern because of its association with high morbidity and mortality due to the liver-related and extrahepatic complications. T2DM represents a common metabolic complication of NAFLD [27]. The prevalence of T2DM in NAFLD patients ranging from 9.8% in mild liver steatosis to 17.8% in moderate to severe liver steatosis [28,29,30]. On the other hand, the proportion of NAFLD among patients with T2DM is ranging between 41.6% and 86%, which is greater than the prevalence in the general population. Recent papers show a complex relationship between NAFLD and T2DM, suggesting a bidirectional and mutual relationship between these two entities [31]. It is well known that NAFLD and T2DM have some identical physiopathological pathways such as IR. Therefore, T2DM can either precede and also promote NAFLD and vice versa. Patients with type 2 diabetes are at higher risk for the development of severe forms of NAFLD, such as non-alcoholic steatohepatitis, advanced fibrosis, and cirrhosis. For instance, in NAFLD the accumulation of triglycerides from toxic metabolites in the liver, muscles, and pancreas can leads to systemic inflammation and IR [4,32,33]. Furthermore, hepatic IR associated with NAFLD is the crucial mechanism for the development of T2DM among these patients. The presence of NAFLD in patients evaluated by the US increases the risk of developing T2DM by 2- to 5-fold [34].

Regarding the screening of NAFLD among diabetic patients, the recommendations are contrary. Thus, European Guidelines suggest that the screening of T2DM in patients with NAFLD can be done by determination of fasting glucose or hemoglobin A1C [35]. Additionally, the American Diabetes Association recommend screening for NAFLD in diabetic patients who have elevated ALT or hepatic steatosis [36]. On the other hand, the American Association Society of Liver Disease do not agree with the routine screening of T2DM patients [37].

US is one of the most commonly available tools for screening, as it is easy to perform and the cheapest. The accuracy of this method for the identification of moderate and severe steatosis was approximately 80% compared to that of liver biopsy in a meta-analysis [38]. The disadvantage of the US is that cannot estimate the grade of steatosis in those with obesity, or those with mild steatosis and depend on the operator. On the contrary, CAP is a quantitative tool for evaluating liver steatosis with higher sensitivity and specificity. Moreover, the histological degree of steatosis correlate with CAP numerical values measurements [39].

The prevalence of severe liver steatosis in our study cohort was 242 (78.5%). In line with these results, Sporea et al. conducted a study that included 776 patients with T2DM and 60.3% of them had severe steatosis [40]. Similar results were found in a study effectuated by Mantovani et al. of 330 patients with type 2 diabetes, where 238 (72.1%) met the diagnostic criteria for NAFLD [30]. Regarding liver fibrosis, in our study, 52 (12.3%) of all patients had F3 and 45 (10.7%) had F4. Our data are consistent with the conclusions of a study conducted in China on a large cohort of 1190 diabetic patients, where 17.7% of them had advanced fibrosis [41]. Additionally, Roulot et al. found that the prevalence of advanced fibrosis in a French cohort of 437 diabetic patients was about 7.3% [12]. A study from Turkey followed our results and showed that liver fibrosis and steatosis were highly common in Turkish patients with T2DM [11]. On the other hand, in the cirrhosis stage, patients present a decreased CAP level; this fact can be explained by the histologically decreasing liver fat content in the setting of cirrhosis. This aspect highlights the clinical importance of quantitative measurements of hepatic steatosis depending on NAFLD fibrosis status, with the possible need for different cut-off values for the CAP score in advanced or cirrhosis fibrosis stages [42].

In our cohort, in the univariate analysis, ferritin, total cholesterol, and HDL-c values were associated with severe steatosis. Moreover, regarding multivariate analysis, BMI, triglycerides, and high blood glucose were independently associated with severe steatosis. Given the results of multivariate analysis, the patients which are predisposed to severe steatosis have hypercholesterolemia and hypertriglyceridemia and they should be selected for screening. Additionally, in a univariate analysis that we performed for LSM values, we found that age, BMI, platelets, and elevated levels of fasting plasma glucose, ALT, AST, and serum uric acid were associated with F3. Regarding multivariate analysis, only age and BMI were independently associated with F3. Thereby, we can conclude that, in our cohort, the group with the highest risk for advanced fibrosis and severe steatosis consists of obese diabetic patients; BMI remains the only independent risk factor that is strongly associated with both CAP and LSM values.

The main prognostic factor in NAFLD patients is the severity of liver fibrosis [43]. When we evaluated fibrosis severity by VCTE in our study cohort, we found that 35.3% of diabetic patients had a high risk of developing severe fibrosis (11.7% had F3 and 13.6% F4), and therefore, they are at risk for the development of portal hypertension, decompensated liver disease, or hepatocellular carcinoma. It is well known that approximately 20% of diabetic patients are at risk for chronic advanced compensated liver disease, and it is necessary to screen all patients with T2DM with liver elastography [44].

Despite data from several studies which showed that high levels of NAFLD’s surrogate markers such as gamma-glutamyltransferase (GGT) and ALT were associated with a high incidence of T2DM, the predictive value of these biological parameters is limited because of the possibility of normal levels in diabetic patients [45,46]. In our study, AST was positively correlated only with LSM. Therefore, VCTE could be a valuable screening modality in diabetic patients. Regarding the important association between BMI and both liver fibrosis and steatosis in our study, VCTE examinations should be especially advisable for patients with T2DM who are overweight or obese. The risk of developing significant or advanced fibrosis among obese T2DM patients is 1.8- and 2.5-fold, respectively comparing with obese individuals without T2DM [47]. Contrary to our study, Chen et al. reported that the association between T2DM with liver steatosis and fibrosis was a more common finding among subjects with an BMI < 25 kg/m^2^. Compared to our results, this highlights the needing for screening hepatic steatosis and fibrosis in all of T2DM patients independent from BMI status [48]. However, one of the problems of implementing VCTE as a modality for screening for NAFLD in T2DM is its costs [49,50].

Our study has some limitations. The first limitation is that the assessment of liver steatosis and fibrosis was performed only non-invasively. LB, which is the gold standard, should be performed. The second limitation was represented by not using LB to evaluate the patients with high levels of AST and advanced fibrosis to identify the patients with NASH. Regarding the cut-off values for fibrosis assessment, we did not use values according to the probe used (M vs. XL). Recent data suggest that if the right probe is used, there are no important differences between liver stiffness measurements obtained by the M and XL probes [51]. Regarding transient elastography limitation, 21 (4%) of the patients were excluded from the final analysis because of their unreliable measurements. We also excluded from the study patients with risk factors for chronic liver disease (hepatitis viruses, chronic infection, using steatogenic drugs, and alcohol abuse).

## 5. Conclusions

In summary, the high prevalence of liver steatosis and fibrosis among patients with T2DM should be a major concern for clinicians. In our cohort, 78.5% of diabetic patients with fatty liver disease had severe steatosis and 13.6% had cirrhosis. Our results suggest that the reciprocal relationship between T2DM and NAFLD leads to the progression of hepatic fibrosis and is a cause to develop liver-related complications with high morbidity and mortality rates. To avoid systemic multilateral damage, it is necessary to screen patients with NAFLD for T2DM, and vice-versa.

## Figures and Tables

**Table 1:** Baseline characteristics of the study group according to the presence of liver steatosis.

	Overall Cohort *n*, 424	Participants with No Steatosis *n*, 116	Participants with Steatosis *n*, 308	*p*-Value
Age (years)	53.67 ± 11.37	48.23 ± 9.62	55.22 ± 10.88	0.021
Females, *n* (%)	219 (51.7)	61 (52.6)	158 (51.3)	0.174
Body mass index (kg/m^2^)	28.33 ± 5.38	26.42 ± 4.47	29.12 ± 5.64	<0.001
Hypertension *n* (%)	246 (58%)	39 (33.6)	207 (67.2)	<0.001
Waist circumference (cm)	105.14 ± 15.2	92.48 ± 12.44	108.21 ± 16.21	<0.001
Platelets × 10^3^/mm^3^	227.23 ± 68.58	205.62 ± 74.31	233.41 ± 72.11	0.022
INR	1.05 ± 0.16	1.02 ± 0.19	1.06 ± 0.17	0.115
Fibrinogen	335.91 ± 57.36	329.63 ± 57.48	339.56 ± 58.22	0.163
Protein-C reactive (mg/dL)	0.65 ± 0.28	0.57 ± 0.24	0.68 ± 0.29	0.24
AST (IU/L)	41.57 ± 23.52	39.05 ± 20.53	42.32 ± 22.92	0.154
ALT (IU/L)	51.46 ± 31.94	42.35 ± 24.31	54.11 ± 30.98	0.034
Total cholesterol (mg/dL)	218.47 ± 58.75	198.81 ± 54.32	226.67 ± 60.43	<0.001
Triglycerides (mg/dL)	191.85 ± 52.62	182.39 ± 39.14	195.74 ± 54.22	<0.001
LDL-c (mg/dL)	117.14 ± 34.54	105.31 ± 30.67	119.28 ± 36.51	<0.001
HDL-c (mg/dL)	48.61 ± 16.89	54.42 ± 19.82	44.31 ± 15.82	0.016
Fasting impaired glucose (mg/dL)	138.78 ± 48.21	118.56 ± 44.78	142.87 ± 49.68	<0.001
Creatinine (mg/dL)	0.89 ± 0.15	0.78 ± 0.14	0.91 ± 0.16	0.054
Urea (mg/dL)	40.51 ± 10.8	38.81 ± 9.97	42.32 ± 10.91	0.072
Serum uric acid (mg/dL)	6.21 ± 1.14	5.78 ± 0.98	6.32 ± 1.16	0.043
Alpha-fetoprotein (ng/mL)	4.61 ± 1.7	4.46 ± 1.22	4.68 ± 1.93	0.084
Lean subjects, *n* (%)	76 (18)	28 (24.1)	48 (15.6)	
Overweight, *n* (%)	175 (41.3)	30 (25.9)	145 (47.1)	
Obese, *n* (%)	173 (40.8)	58 (50)	115 (37.3)	
Steatosis degree, *n* (%)				<0.001
0	116 (27.4)		0	
1	40 (9.4)		40 (13.1)	
2	26 (6.1)		26 (8.4)	
3	242 (57.1)		242 (78.5)	
Fibrosis stage, *n* (%)				<0.001
0	182 (43)	51 (43.9)	131 (42.5)	
1	110 (25.9)	32 (27.6)	78 (25.3)	
2	34 (8.1)	14 (12.1)	20 (6.5)	
3	53 (12.5)	16 (13.8)	36 (11.7)	
4	45 (10.7)	3 (2.6)	42 (13.6)	
CAP, dB/m	314.11 ± 49.54	255.41 ± 18.38	317.42 ± 50.98	<0.001
LSM, kPa	7.48 ± 4.11	6.32 ± 4.74	8.02 ± 5.16	<0.001

INR, international normalized ratio; ALT, alanine aminotransferase; AST, aspartate aminotransferase; LDL-c, low-density lipoprotein cholesterol; HDL-c, high-density lipoprotein cholesterol; LSM, liver stiffness measurement; CAP, controlled attenuation parameter.

**Table 2:** Characteristics of patients according to liver fibrosis stage.

	≤F2 *n*, 326	≥F3 *n*, 98	*p*
Age (years)	50.88 ± 15.52	55.15 ± 12.24	0.041
Females, *n* (%)	169 (51.8)	50 (51)	0.066
Body mass index (kg/m^2^)	25.75 ± 3.51	28.07 ± 3.22	<0.001
Lean subjects, *n* (%)	58 (17.8)	18 (18.3)	0.296
Overweight, *n* (%)	152 (46.6)	23 (23.5)	0.016
Obese, *n* (%)	116 (35.6)	57 (58.2)	0.008
Hypertension, *n* (%)	197 (60.4)	49 (50)	0.072
Platelet count (G/L)	254.42 ± 73.31	191.9 ± 74.81	<0.001
ALT (IU/L)	32.68 ± 26.23	39.19 ± 24.02	0.133
AST (IU/L)	27.71 ± 12.37	40.87 ± 22.43	<0.001
GGT (IU/L)	49.49 ± 19.46	50 ± 20.68	0.061
ALP (IU/L)	73.95 ± 26.02	78.38 ± 39.43	0.052
Total bilirubin (mg/dL)	0.76 ± 0.31	0.84 ± 1.12	0.084
Albumin (g/dL)	4.57 ± 0.4	4.1 ± 0.57	<0.001
Creatinine (mg/dL)	0.79 ± 0.15	0.84 ± 0.19	0.121
Urea (mg/dL)	35.8 ± 10.35	37.02 ± 15.5	0.082
Fasting glucose (mg/dL)	106.15 ± 30.08	119.36 ± 26.04	0.024
Ferritin (ng/mL)	174.6 ± 110.28	187.46 ± 183.7	0.416
CRP (mg/dL)	0.42 ± 0.52	0.46 ± 1.12	0.093
Total cholesterol (mg/dL)	207.9 ± 49.41	224.1 ± 54.9	0.072
Triglycerides (mg/dL)	198.81 ± 77.58	213.29 ± 65.27	0.047
LDL-c (mg/dL)	116.28 ± 49.01	118.12 ± 49.63	0.098
HDL-c (mg/dL)	46.7 ± 13.7	41 ± 12.03	0.004
Serum uric acid (mg/dL)	4.86 ± 1.6	6.09 ± 1.51	<0.001
Alpha-fetoprotein (ng/mL)	3.93 ± 2.29	5.19 ± 4.15	0.021
Steatosis degree			0.008
0	107 (32.8)	9 (9.2)	
1	29 (8.9)	11 (11.2)	
2	14 (4.3)	12 (12.3)	
3	176 (54)	66 (67.3)	
Fibrosis stage, *n* (%)			
0	182 (55.8)	-	
1	110 (33.7)	-	
2	34 (10.5)	-	
3	-	53 (54.1)	
4	-	45 (45.9)	
CAP, dB/m	308.79 ± 40.65	319.54 ± 45.47	0.011
LSM, kPa	6.47 ± 4.27	11.53 ± 5.98	<0.001
M-probe, *n* (%)	238 (73)	61 (62.2)	0.059
XL-probe, *n* (%)	88 (27)	37 (37.8)	0.048

ALT, alanine aminotransferase; AST, aspartate aminotransferase; GGT, gamma-glutamyl transferase; ALP, alkaline phosphatase; LDL-c, low-density lipoprotein cholesterol; HDL-c, high-density lipoprotein cholesterol; LSM, liver stiffness measurement; CAP, controlled attenuation parameter; CRP, c-reactive protein.

**Table 3:** Univariate and multivariate linear regression analysis of factors associated with increased LSM values.

	LSM kPa			
	Univariate		Multivariate	
Parameter	Beta	*p*-Value	Beta	*p*-Value
Age	0.123	0.004	0.157	0.013
BMI	−0.096	<0.001	0.142	<0.006
CAP	0.034	0.014		
Platelets	−0.307	<0.001	−0.124	0.073
Fibrinogen	−0.110	0.138		
INR	0.167	0.123		
CRP	0.097	0.192		
Ferritin	0.069	0.355		
FPG	0.121	0.002	0.136	0.011
Urea	−0.046	0.534		
Creatinine	0.008	0.915		
ALT	0.297	<0.001	0.085	0.332
AST	0.306	<0.001	0.108	0.227
Cholesterol	0.047	0.525		
Triglycerides	0.177	0.001	0.128	0.021
Albumin	−0.061	0.414		
LDL-c	−0.047	0.529		
HDL-c	−0.084	0.029	0.076	0.062
SUA	−0.17	0.016	0.182	0.058
AFP	0.431	0.051		

BMI, body mass index; ALT, alanine aminotransferase; AST, aspartate aminotransferase; LDL-c, low-density lipoprotein cholesterol; HDL-c, high-density lipoprotein cholesterol; CAP, controlled attenuation parameter; CRP, c-reactive protein; INR, international normalized ratio; AFP, alpha-fetoprotein; SUA, serum uric acid, FPG, fasting plasma glucose.

**Table 4:** Univariate and multivariate linear regression analysis of factors associated with an increased CAP score.

	CAP dB/m			
	Univariate		Multivariate	
Parameter	Beta	*p*-Value	Beta	*p*-Value
Age	0.045	0.540		
BMI	0.393	<0.001	0.349	<0.001
CAP	-	-	-	-
Platelets	−0.003	0.963		
Fibrinogen	0.042	0.574		
INR	−0.148	0.145		
CRP	0.164	0.126		
Ferritin	0.223	0.022	0.174	0.059
FPG	0.255	0.001	0.211	0.006
Urea	0.137	0.064		
Creatinine	0.013	0.860		
ALT	0.086	0.243		
AST	0.061	0.409		
Cholesterol	−0.159	0.031	−0.122	0.035
Triglycerides	−0.126	0.018	−0.132	0.044
Albumin	0.118	0.112		
LDL-c	−0.011	0.881		
HDL-c	0.120	0.006	−0.113	0.032
SUA	0.079	0.285		
AFP	−0.042	0.571		

BMI, body mass index; ALT, alanine aminotransferase; AST, aspartate aminotransferase; LDL-c, low-density lipoprotein cholesterol; HDL-c, high-density lipoprotein cholesterol; CAP, controlled attenuation parameter; CRP, c-reactive protein; INR, international normalized ratio; AFP, alpha-fetoprotein; SUA, serum uric acid, FPG, fasting plasma glucose.

**Table 5:** Comparison of LSM and CAP values according to fibrosis stages.

	LSM kPa	*p*-Value	CAP dB/m	*p*-Value
F0	4.62 ± 0.76	<0.001	297 ± 44.88	0.043
F1	7.10 ± 0.85		314 ± 46.88	
F2	8.85 ± 0.47		311 ± 46.6	
F3	11.22 ± 1.21		317 ± 47.28	
F4	21.55 ± 7.5		304 ± 63.23	

## Data Availability

The data presented in this study are available on request from the corresponding author. The data are not publicly available because they are the property of the Institute of Gastroenterology and Hepatology, Iasi, Romania.
